# A Binary Salt Mixture LiCl–LiOH for Thermal Energy Storage

**DOI:** 10.3390/ma16041434

**Published:** 2023-02-08

**Authors:** Naveed Hassan, Manickam Minakshi, John Ruprecht, Willey Yun Hsien Liew, Zhong-Tao Jiang

**Affiliations:** 1 Surface Analysis and Materials Engineering Research Group, College of Science, Health, Engineering and Education, Murdoch University, Perth, WA 6150, Australia; 2 College of Science, Health, Engineering and Education, Murdoch University, Perth, WA 6150, Australia; 3 Centre for Energy, Water, and Waste, Harry Butler Institute, Murdoch University, Perth, WA 6150, Australia; 4 Faculty of Engineering, Universiti Malaysia Sabah, Jalan UMS, Kota Kinabalu 88400, Malaysia

**Keywords:** phase change materials, eutectic LiCl–LiOH salt, thermal stability, thermal energy storage

## Abstract

For thermal energy storage, the most promising method that has been considered is latent heat storage associated with molten salt mixtures as phase-change material (PCM). The binary salt mixture lithium chloride—lithium hydroxide (LiCl–LiOH) with a specific composition can store thermal energy. However, to the best of our knowledge, there is no information on their thermal stability in previous literature. The key objectives of this article were to investigate the thermophysical properties, thermal repeatability, and thermal decomposition behavior of the chosen binary salt mixture. FactSage software was used to determine the composition of the binary salt mixture. Thermophysical properties were investigated with a simultaneous thermal analyzer (STA). The thermal results show that the binary salt 32 mol% LiCl-68 mol% LiOH melts within the range of 269 °C to 292 °C and its heat of fusion is 379 J/g. Thermal repeatability was tested with a thermogravimetric analyzer (TGA) for 30 heating and cooling cycles, which resulted in little change to the melting temperature and heat of fusion. Thermal decomposition analysis indicated negligible weight loss until 500 °C and showed good thermal stability. Chemical and structural instability was verified by X-ray diffraction (XRD) by analysing the binary salt system before and after thermal treatment. A minor peak corresponding to lithium oxide was observed in the sample decomposed at 700 °C which resulted from the decomposition of LiOH at high temperature. The morphology and elemental distribution examinations of the binary salt mixture were carried out via scanning electron microscopy (SEM) coupled with energy dispersive spectroscopy (EDS). X-ray photoelectron spectroscopy was conducted for surface analysis, and their elemental composition verified the chemical stability of the binary salt mixture. Overall, the results confirmed that the binary salt mixture is a potential candidate to be used as thermal energy storage material in energy storage applications of up to 500 °C.

## 1. Introduction

The foremost problems that human beings currently face are concerns about energy and the environment. Energy demand has increased massively in the last few decades due to a boom in industry and an increase in the global population. Fossil fuels have been the world’s main source of energy; however, it has caused severe environmental impacts due to greenhouse gas emissions [1,2,3]. These concerns make a compelling case for the utilization of alternative energy, and the transition to alternative energy poses a central question of reliability and other challenges. Researchers have started to work on alternative renewable energy resources to slow down climate change and yield sustainable developments. Solar, wind, and hydro are the most widely used renewable energy sources. Among these, solar energy is adequate for fulfilling the current energy consumption requirements. However, its storage is a pivotal factor to take into consideration, especially for night and off-peak periods when there is no or minimal sunlight [4,5,6]. Currently, the majority of research is dedicated to discovering materials for efficient heat energy storage.

Phase change materials (PCMs) are the most attractive materials used in latent heat thermal energy storage (TES) systems to absorb or release heat energy during phase transition. PCMs provide realistic and improved storage efficiency in TES systems. Additionally, the specific heat of a latent heat TES system is 50–100 times greater than that in the sensible heat TES system [2,7,8]. When selecting PCMs for the TES system, the main factors to consider are melting temperature, heat of fusion, and economic cost. Among PCMs, salts and their mixtures have wide melting temperature ranges and high heating enthalpies; therefore, they are most suitable to be used in latent heat TES systems [9]. As the thermal properties of pure salts are fixed, to fulfill the requirements of any TES system, the thermal characteristics of pure salts can be modified by making their mixtures at different compositions. Phase temperature range can also be expanded by making appropriate salt mixtures. The major drawbacks of salts and their mixtures are low thermal conductivity and corrosion [10,11]. Salts of fluoride, chloride, carbonate, nitrate, and hydroxide have been proposed as possible PCMs in previous literature [3,12]. Nitrate salts have a low melting temperature whereas hydroxides melt between 250 °C and 600 °C. Fluoride, carbonate, and chloride salts melt above 600 °C [1]. Lithium compounds were investigated in [12] and revealed that a high number of compounds have enthalpies above 200 kJ/kg, which heightens the favourableness of lithium compounds as PCM. Lithium has been employed widely to improve the characteristics of molten salts used today. Lithium-containing salts are mostly hygroscopic, and absorbing moisture from the air is also a problem. While absorbing moisture, the salt will lose its chemical stability and in turn, the material’s heat storage capacity would be lost. During the selection of material for TES applications, this property should be considered. It was also also proposed by study [12] that lithium salts LiCl, LiOH, and LiNO_3_ as the most attractive materials for latent heat storage systems. Nevertheless, more insights into their thermal and cycling stability are required. In addition to single lithium salts, binary and ternary salt mixtures also present attractive latent heat values of over 130 kJ/kg, within a wide range of melting temperatures (76–770 °C). This allows the utilization of these compounds for low, medium, and high-temperature applications. To mention a few of them, LiNO_3_-urea and LiCl–AlCl_3_ are the only proposed binary PCMs that can be applied for low-temperature storage, both with latent heat near 200 kJ/kg. Na_2_CO_3_-Li_2_CO_3_ (58–42 wt%) was characterized in [13] using various techniques such as DSC, TGA, and XRD. In which, DSC and TGA tests were held from temperature ambient to 600 °C with 10 °C /min heating/cooling rate in CO_2_ and N_2_ atmospheres. The heat of fusion and solidification were 330.8 ± 0.6 kJ/kg and 329.2 ± 0.3 kJ/kg respectively, which were quite similar. The measured temperature of melting was 498.3 ± 0.1 °C higher than its solidification points by 14.4 ± 0.2 °C, indicating the sub-cooling. It performed well in reversibility for 500 thermal cycles and showed good thermal and chemical stability without any loss in weight under a CO_2_ environment, and a 0.8% weight loss was detected in an N_2_ atmosphere. Another eutectic salt, LiNO_3_-NaCl (87–13 wt%), was characterized in a study [14] using similar techniques for their thermo-physical properties and cycling stability. The material was tested for cycling stability and heated 51 times between 50 and 250 °C at the rate of 10 °C/min. The melting point measurement during 51 cycles showed deviations from the average value within ±0.4%, and latent heat showed deviations within ±1.5%, which are acceptable for use as a PCM. Also, weight loss was 0.26% recorded for 50 cycles. Thermal decomposition temperature was determined between 400 °C and 450 °C, as NaCl can withstand high temperatures; however, LiNO_3_ decomposes at this temperature. Two new ternary eutectic salt mixtures, fluoride salts LiF-MgF_2_-KF (64–30–6 wt%) and carbonate salts Li_2_CO_3_-K_2_CO_3_-Na_2_CO_3_ (32.1–34.5–33.4 wt%) filled in a cylindrical container, showed no phase separation or subcooling over 50 thermal cycles. In addition, cycling stability was also tested for this mixture of Li_2_CO_3_-K_2_CO_3_-Na_2_CO_3_, exhibiting 1000 thermal cycles [15,16].

Lithium salts and their eutectic mixtures are used in many applications because of their suitable melting temperature range and high fusion enthalpies. Lithium salts and eutectics with a range of melting temperatures of 0–100 °C are mostly used for solar-assisted air conditioning systems [17]. Few eutectic salts are reported with a temperature range of 100–400 °C are proposed for applications such as heated underwater diver suits and portable thermal conditioning jackets. High-temperature lithium-based salts with a melting temperature range from 400–1100 °C are proposed for solar receiver space applications, Stirling engine, and heat-cold prototypes [12,18]. Moreover, these salts gained much attention to be used in advanced solar dynamic power systems as alternatives to batteries and heavy fuel cells [19,20]. Due to the larger consumption of lithium-containing salts in applications, a significant increase in demand for lithium-containing salts is anticipated in the coming years. The forecast of lithium-containing salts demand by compound and consumption by application is shown in Table 1. Lithium is mostly produced as a mineral in Australia, Canada, Africa, Russia, and Brazil. It is also produced as brine in Chile, United States, China, and Argentina. Approximately 62% of the world’s resources of lithium are from brines [21,22]. In 2010, 25,300 t were produced annually, in which 35% were produced by Chile, 33% by Australia, 18% by China, and the remaining 14% by other countries [23].

Thus, lithium salts are the potential candidates for thermal energy storage with promising thermal properties and thermal stability. However, there is very limited research work done on determining the thermal and chemical stability. Due to their suitable melting temperature, high heat of fusion, and good thermal stability, the mixture of lithium salts (LiCl–LiOH) is selected for the current study. The eutectic composition (37 mol% LiCl-63 mol% LiOH) was investigated in [25] only for their thermal properties. The melting temperature and heat of fusion were reported as 258 °C and 485 J/g, respectively. However, thermal, and chemical stability has not been reported in earlier literature. The main objective of this paper is to investigate the thermal properties of salt mixtures in different compositions, thermal stability, thermal reliability, and chemical stability. As these properties play a vital role in the selection of energy storage materials for any energy storage application. Thermophysical properties such as melting temperature, solidification temperature, the heat of fusion, and heat of solidification were determined by a simultaneous thermal analyzer (STA). Thermal stability, thermal reliability, and chemical stability were investigated by a thermogravimetric analyzer (TGA). X-ray diffraction (XRD), scanning electron microscopy (SEM) along with energy dispersive spectroscopy (EDS), and X-ray photoelectron spectroscopy (XPS) was used for verification of the chemical stability of the salt mixture. Thus, this work provides insight into the thermal stability, thermal reliability, and chemical stability of the LiCl–LiOH salt mixture.

## 2. Experimental and Methodology

### 2.1. Sample Preparation

The mixture of the two lithium salts can be prepared by direct and indirect mixing [26]. Most of the researchers used the direct method, in which the two salts were mixed in a solid-state mixture. Indirect mixing, the materials mixed in deionized water, has not been widely reported previously, and the salt might react with water and influence their properties.

Both the chemicals LiCl and LiOH were provided by Sigma Aldrich with greater than 99% purity. Before mixing, both chemicals were kept in the oven at 100 °C for 60 min to completely evaporate any trapped moisture. This was done to obtain the most precise composition of the binary salt mixture. After this process, each chemical with the specific mole composition was weighed rapidly to avoid any moisture capture. Each salt with the required mole composition was mixed well in an alumina crucible and heated in a furnace starting at 50 °C up to 350 °C at a 5 °C/min heating rate. The mixture was then held at this temperature for 40 min to ensure the uniform composition of the salt. Finally, the temperature of the mixture was ramped down to room temperature. The final powdered form was kept in a sealed tight desiccator for further examination. For this project, four lithium salt mixtures at different compositions were made and tabulated in Table 2 with their respective lithium chloride (LiCl) and lithium hydroxide (LiOH) compositions as stated. We chose 37 mol% LiCl-63 mol% LiOH, the eutectic composition of the binary salt, from the literature [25] and a phase diagram obtained from the FactSage [27]. While the compositions of the other three samples were chosen randomly with a difference of 5 mol% from each other.

### 2.2. Materials Characterization

The thermal characterization of the binary salt mixture was done via a simultaneous thermal analyzer (PerkinElmer, STA/TGA-8000) operated on the Pyrus software system. This was employed to determine its onset melting temperature, solidification temperature, the heat of fusion, and the heat of solidification. To investigate the thermal repeatability and stability of the salt mixture, STA was used in the mode of TGA (thermogravimetric analyzer). The maximum temperature up to which the STA/TGA-8000 worked accurately ranged from 25 to 1600 °C. Argon, nitrogen, or air was used as a purging medium with a flow rate of 200 mL/min at maximum. In this instrument, a ceramic pan with a net sample mass of 10–1000 mg with a recommendation of 10–20 mg was used for precision in experimental results. The heating and cooling ramps could be adjusted from 0.1–100 °C/min. The STA was calibrated by using gold as shown in Table 3. The error in melting temperature was measured as 0.2%; however, for measuring enthalpy, the 3% relative error was calculated. Individual component LiCl was also tested for melting temperature and heat of fusion, and a 1% relative error was recorded. So, taking those results into account, the instrument is reliable.

For verification of the chemical stability of the mixtures, an X-ray diffraction instrument (Rigaku XRD) was used. Scanning electron microscopy SEM (SEM, Zeiss Neon 40EsB) was used for viewing the morphology of the prepared binary salt mixtures. Meanwhile, energy dispersive spectroscopy (EDS) was used to analyze the elemental distribution. For surface analysis and quantification, the X-ray photoelectron spectroscopy XPS was performed on Kratos AXIS Ultra DLD spectroscopy (Shimadzu Corp., Tokyo, Japan) using Al (hν = 1486.7 eV) or Ag (hν = 2984.3 eV) X-ray sources in ultra-high vacuum for the determination of elemental and composition of samples.

### 2.3. Experimental Procedure

By using STA-8000, the thermophysical properties such as melting temperature, solidification temperature, fusion enthalpy, and heat of solidification were obtained. Samples were packed with an Al_2_O_3_ crucible for all measurements, and the sample quantity for each test was about 10–15 mg. During the experiment, the samples were constantly heated from 50–350 °C and cooled from 350–50 °C keeping the heating ramps at 10 °C/min, purging with argon gas at a constant flow rate of 20 mL/min. The sample was brought back to 50 °C at the same rate (10 °C/min). To minimise, the effect of absorbed moisture in the initial cycle, the procedure was repeated eight times. The heat flow vs. temperature curve was plotted, and the thermophysical properties were determined.

To test the thermal repeatability of the binary salt mixture, repeated cycles were performed in the STA-8000 analyser. The heating and cooling of the sample, approximately 15 mg, was repeated 30 times under the same conditions as for thermophysical properties. The latent heat and transition temperature of each cycle were measured. By doing so, cycling stability was also achieved by obtaining the weight loss of the binary salt mixture during these 30 cycles. The chemical stability along with thermal decomposition was evaluated with the help of TGA. The mixture was heated up from 50–350 °C and cooled to 50 °C in an alumina crucible without a lid under similar conditions used for finding thermophysical properties to allow evaporation of any moisture. By doing so, the sample was heated again, but from 50–700 °C, while the rest of the conditions and heating rates were kept as such, and the decomposition temperature was measured from weight loss. To identify the changes in the crystalline phases, the samples in powder form were evaluated in XRD with 2θ scan from 20° to 90° and a step size of 0.013. SEM/EDS was used to examine the morphology and elemental distribution of the thermally cycled samples, from which the chemical stability was verified. While preparing samples for SEM, it was soon converted into liquid as samples were hygroscopic and very sensitive to air. So, instead of the conventional way of preparing samples, a special holder was used in which samples remained in an inert atmosphere to avoid moisture capturing from the air. Samples were prepared in the glove box in an argon atmosphere by putting all the stubs in the special holder inside the glove box and brought to SEM for characterization. All the samples were run under 10 kV.

The surface properties such as elements and their compositions of the binary salt mixture were characterized by XPS, using a monochromated Al Kα X-ray source (1486.7 eV) with an analyzer pass energy of 160 eV for survey scans and 20 eV for detailed elemental scans. For the analysis of data received from the XPS, software called CASAxps and NIST database [28] (NIST, 2000, 6 June) was used. As samples were highly sensitive to air, samples were mounted on the holder by carbon tape and transferred inside the XPS chamber quickly. By doing so, moisture absorption was avoided.

## 3. Results and Discussions

### 3.1. Phase Diagram, Melting Temperature, and Heat of Fusion

**Figure 1:**
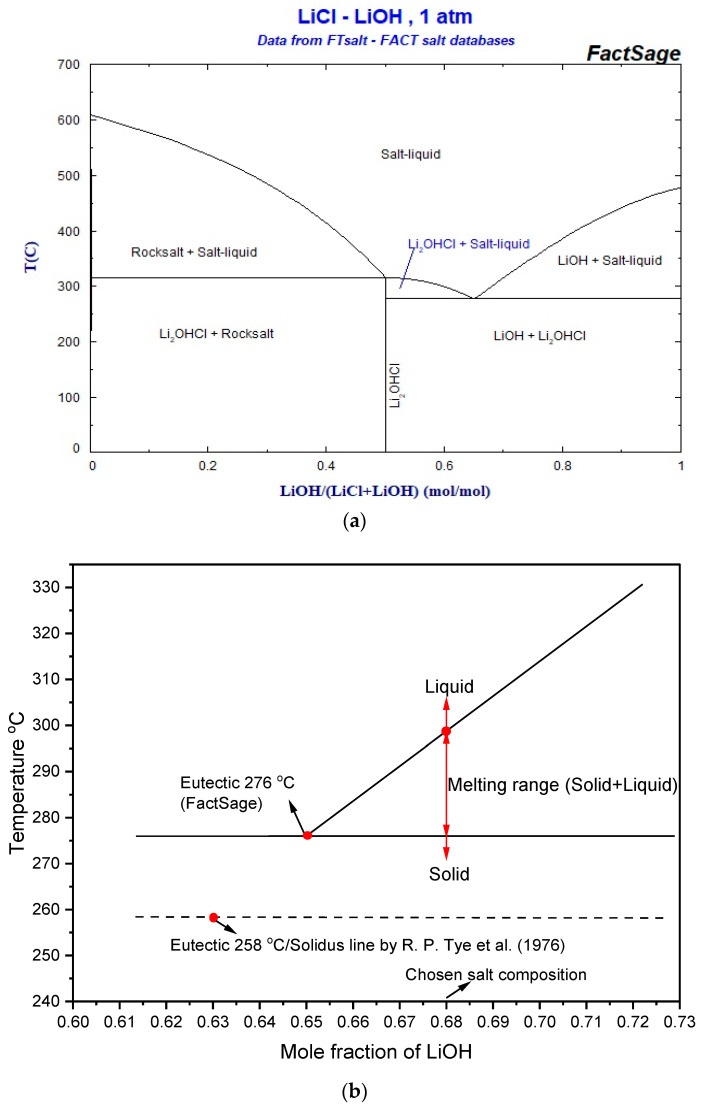
(**a**) Phase Diagram of eutectic binary mixture LiCl–LiOH obtained from FactSage (FactSage, 28 March 2022). (**b**) Phase diagram of investigated binary salt mixture 32 mol% LiCl-68 mol% LiOH derived from FactSage tool and Ref. [25].

The phase diagram of the eutectic salt, taken from the FactSage tool is shown in Figure 1a. The result from FactSage (FactSage, 28 March 2022) indicated eutectic composition of 37 mol% LiCl and 63 mol% LiOH exhibited at a temperature of 262 °C. Thermophysical properties of 37 mol%–63 mol% were investigated in literature [25] experimentally. The report showed the melting temperature was 258 °C and the heat of fusion was 485 J/g. In the current study, 37 mol% LiCl–63 mol% LiOH was tested for their thermal properties. It was observed that the average melting temperature was 270 °C, which is closer to the reported values by Tye et al. However, the average heat of fusion of the samples was 297 J/g which was significantly different from the values reported in literature [25]. The observed difference could be due to the accuracy and reliability of the equipment in 1976, which might have affected their results. Therefore, the composition of the material was altered in our studies with a difference of 5 mol%, and we prepared the other three samples (labelled as 1, 2, and 4 in Table 4).

Considering that the phase transition properties can be impacted by the heating/cooling ramps, the salt mixture was tested initially at different heating/cooling rates, and the chosen rate (10 °C/min) resulted in adequate properties. The melting temperature and heat of fusion of the four samples are given in Table 4. Among the four samples studied, the most suitable sample was found to be 32 mol% LiCl-68 mol% LiOH, which was selected for our further experiments resulting from its higher heat of fusion and suitable melting temperature. The phase diagram of the chosen binary salt mixture (32 mol% LiCl-68 mol% LiOH) derived from the FactSage FTSalt database and R. P. Tye et al. (1976) is shown in Figure 1b.

**Figure 2:**
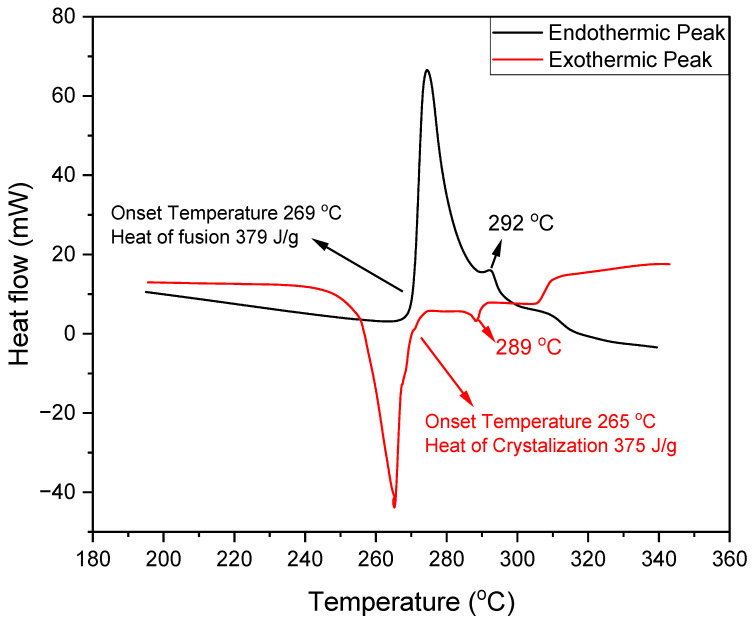
STA curve of 32 mol% LiCl-68 mol% LiOH indicating the melting temperature range and heat of fusion values.

Figure 2 revealed the STA curve of the binary salt mixture which displayed two peaks in each curve (endothermic and exothermic curve), indicating that the binary salt mixture was homogenous and non-eutectic. The composition of the binary salt mixture (32 mol% LiCl-68 mol% LiOH) was very close to the eutectic composition shown by FactSage and reported in literature [25]; therefore, the two peaks in each curve were very close to each other. Due to non-eutectic behavior, the salt mixture melted in the range of temperature with a starting melting temperature (solidus) of 269 °C and an ending melting temperature (liquidus) of 292 °C. Similarly, the salt mixture solidified within the range of 289 °C to 265 °C. Comparing the STA curves with the phase diagram in Figure 1b, the binary salt mixture melted and solidified within the range of the reported values. Thus, the results in this work agreed with FactSage and literature [25]. The enthalpy of fusion of this mixture was 379 J/g, and the heat of solidification was 375 J/g. The little difference between these values could have also been the result of evaporation. Creeping could also be the possible reason, as some salts, mostly chloride salts, creep towards the wall of the crucible during heating after several cycles during STA measurement, which could possibly lower the heat of fusion of the sample.

### 3.2. Thermal Repeatability

The thermal repeatability of the PCM is a crucial parameter for determining its long-term stability through many cycles. After several cycles, melting and solidification of a PCM must be able to occur with a constant phase change in temperature and latent heat. In this experiment, the salt mixture (32 mol % LiCl–68 mol % LiOH) was repeatedly heated and cooled in the argon atmosphere, between 50 and 350 °C at a rate of 10 °C/min, for 30 times. The salt could absorb moisture (hygroscopic) and be soluble in water. During sample preparation, precautions were taken to control the moisture absorption, or else it would risk errors in weight calculation, which in turn could impact the outcomes of the experiment. As a result, the initial cycles in which the moisture was driven were discounted from all the data.

**Figure 3:**
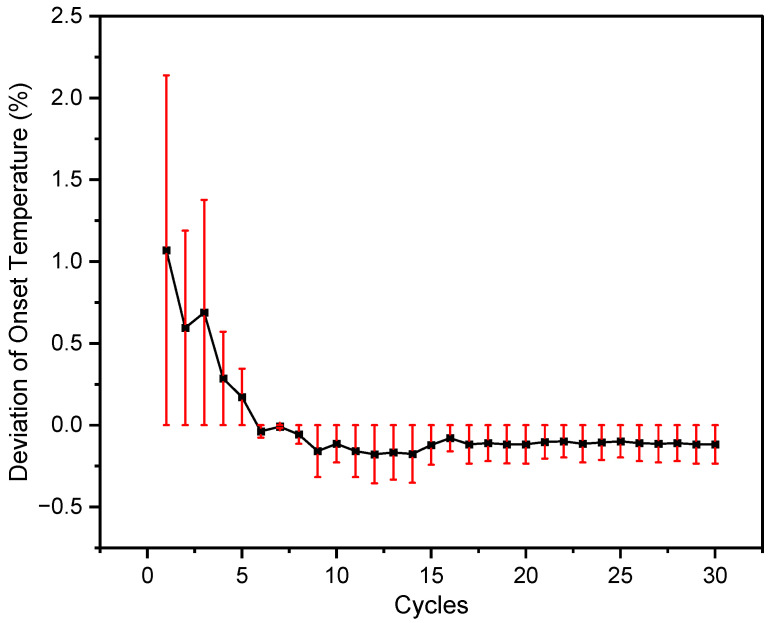
Deviation of melting temperature with error bars illustrated for each cycle from the average calculated value for the best-chosen sample i.e., 32 mol% LiCl-68 mol% LiOH.

**Figure 4:**
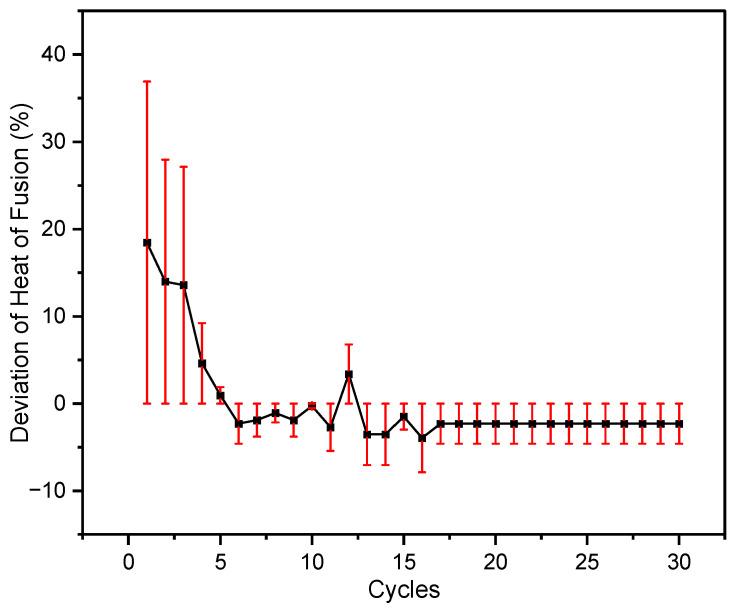
Deviation of heat of fusion with error bars illustrated for each cycle from the average calculated value for the best-chosen sample, i.e., 32 mol% LiCl-68 mol% LiOH.

To calculate the deviations of the measured melting temperature and latent heat from their average value, Equations (1) and (2) were used, shown in Figure 3 and Figure 4. The average heat of fusion of the 30 heating/cooling cycles was 345 J/g. The melting temperature and heat of fusion of the first cycle were 268 °C and 391 J/g, respectively. Whereas for the 30th cycle, the melting temperature was recorded as 265 °C and the heat of fusion was 340 J/g. The melting temperature of the binary salt was consistently nearly the same with a small deviation of within ±0.2% from the average value, excluding the initial three cycles. Apart from the first few cycles, the maximum error in melting temperature was less than ±0.2, showed little variation between the cycles, and indicated excellent repeatability. For latent heat, the deviation between the measured and average values was ±3%, discounting the first few cycles. Moreover, the maximum error determined after the first four cycles was ±3. These deviations and errors could be due to the weight loss by the evolution of gases during repeated heating. Creeping could also be the reason which can result in a reduction of the heat of fusion after several cycles. Thus, from the above results, the molten salt mixture appeared to be reliable thermal energy storage material.

(1) $$T_{\mathrm{deviation}}=\frac{T_{n}-{\;T}_{\mathrm{avg}}}{T_{\mathrm{avg}}}\;1\;\leq n\;\leq 30$$

(2) $$\Delta H_{\mathrm{deviation}}=\frac{\Delta H_{n}-\Delta H_{\mathrm{avg}}}{\Delta H_{\mathrm{avg}}}\;1\;\leq n\;\leq 30\;$$

T_deviation_ and ∆H_deviation_ is the deviated value of melting temperature and heat of fusion, respectively. T_n_ and ∆H_n_ is the melting temperature and heat of fusion of each cycle, respectively. T_avg_ and ∆H_avg_ are the average melting temperature and average heat of fusion of all 30 cycles, respectively, whereas n is the cycle number.

### 3.3. Thermal Stability

**Figure 5:**
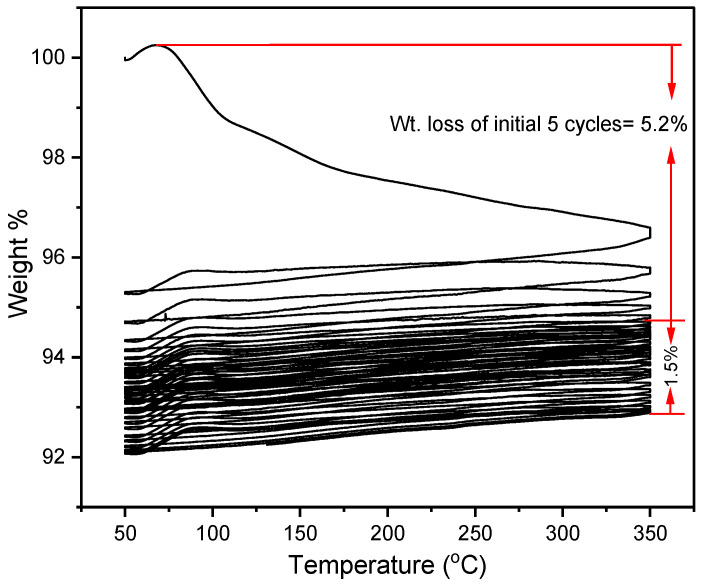
Weight loss vs. temperature plot of chosen salt composition (32 mol% LiCl-68 mol% LiOH) for 30 cycles.

If the binary salt is thermally stable in the range of 120 °C to 350 °C, which is a pre-requisite, a very large quantity of heat could be available. Thermal stability was tested with the help of TGA, under similar conditions to that of other experiments. As seen in Figure 5, the significant weight loss in the first five cycles was due to the hygroscopic characteristics of the binary salt mixture. The initial weight loss is attributed to the loss of trapped moisture in the sample. As compared to the first five thermal cycles, the other 25 cycles resulted in minimal weight loss.

**Figure 6:**
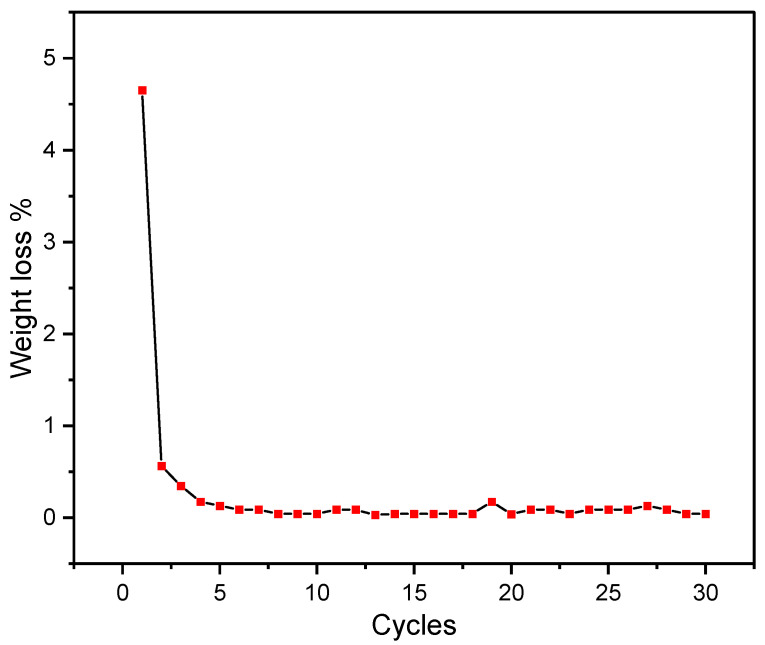
Weight loss % of 32 mol% LiCl-68 mol% LiOH shown for each cycle.

The weight loss percentage for every cycle is displayed in Figure 6. Thermal stability of the binary salt was very significant as the weight loss that occurred in some cycles is minor except for the first few cycles. In some cycles, a very negligible weight gain can be observed, possibly due to the absorption of moisture in the argon atmosphere, if it was not completely dry, as LiCl in the mixture has significant moisture-absorption qualities. Further possible reasons could be due to the accuracy of TGA. The weight loss of binary salt in the first five cycles was 5.2%, and after the 5th cycle, the total weight loss was 1.5%, which was satisfactory. In terms of thermal stability, the results of these trials further indicated the viability of the binary salt for thermal energy storage applications within 350 °C.

### 3.4. Chemical/Structural Stability

**Figure 7:**
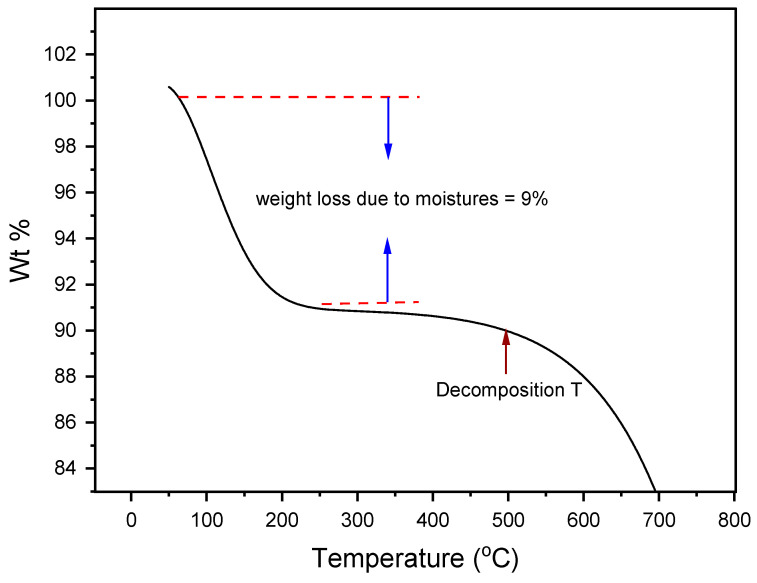
Decomposition profile of chosen salt composition (32 mol% LiCl-68 mol% LiOH).

It is pivotal to test the thermal decomposition behavior of a PCM as they are intended for thermal storage applications. Above the threshold value of the set temperature, the PCM will lose its initial thermal characteristics, and practically all decomposition processes are irreversible. To evaluate the temperature at which decomposition would start, a stability test at a higher temperature was carried out with the help of TGA. For weight loss measurement, the mixture was heated from 50–700 °C with a heating ramp of 10 °C/min. It is clearly observed from Figure 7 that heating up to 130 °C resulted in a huge weight loss which is because of the absorbed moisture. When heating up to 500 °C, the weight loss was minimal and could be a safe window, after this a huge weight loss occurred. This means that binary salt started decomposition and converted into gasses at 500 °C. Based on the results, it was determined that LiCl was stable even after 500 °C; however, LiOH was not a stable compound and it decomposed thermally after 462 °C, producing lithium oxide and water [29].

**Figure 8:**
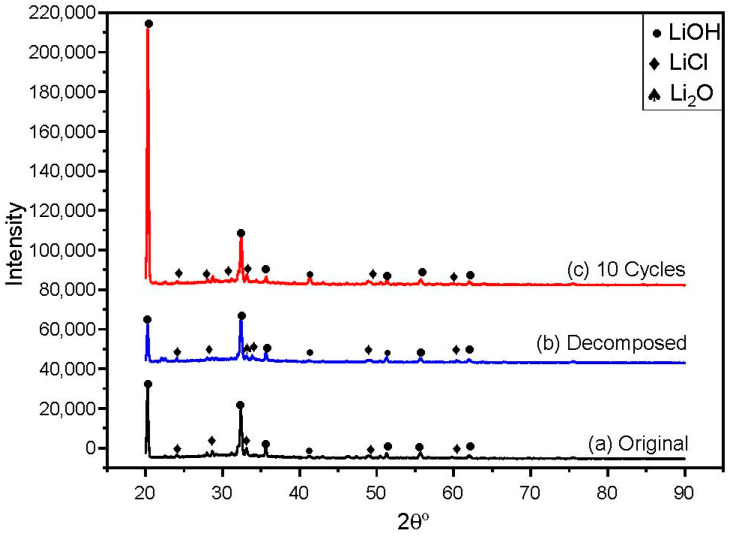
XRD patterns of the chosen salt composition (32 mol% LiCl-68 mol% LiOH) at different conditions (**a**) as prepared, (**b**) heated at 700 °C, and (**c**) cooled for 10 cycles.

The thermal/structural instability was determined by examining the best-chosen samples in XRD to identify if any new phases were evolving under different conditions. It can be seen in Figure 8 that all three binary lithium salt forms uniform phases which are identified to be a parent compound. The three patterns obtained with the help of XRD were alike but the intensity of LiOH varied a little for each sample tested at different conditions, indicating no apparent phase change of the salt mixture occurred for the samples decomposed at 700 °C and after 10 thermal cycles. However, very small peaks of Li_2_O were observed in the sample decomposed at 700 °C, which could have resulted from the decomposition of LiOH in the sample at a higher temperature. It is therefore concluded that LiCl–LiOH did not react with each other and showed good thermal stability.

### 3.5. SEM/EDS Characterization

**Figure 9:**
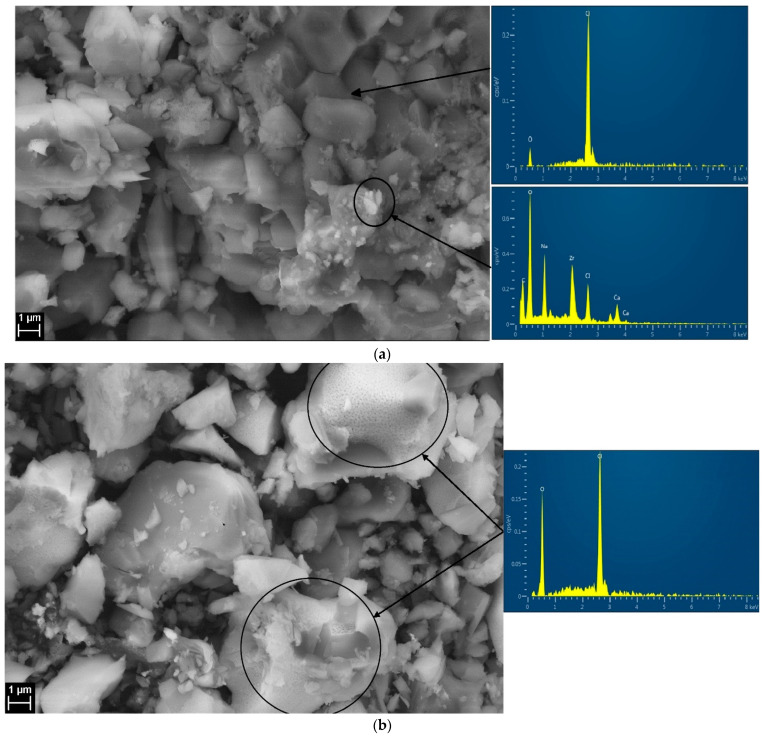
(**a**) SEM/EDS of 32 mol% LiCl-68 mol% LiOH as prepared with zero thermal cycles. (**b**) SEM/EDS of 32 mol% LiCl-68 mol% LiOH decomposed at 700 °C.

A concentration of 32 mol% LiCl-68 mol% LiOH with zero thermal cycles and decomposed at 700 °C was investigated by SEM/EDS to check the morphology and chemical stability of the binary salt, and their micrographs along with the EDS graphs are shown in Figure 9a,b. The micrographs indicated that the particles of both salts are evenly distributed and densely packed. It was observed that there was a small change in the particle posture of the sample prior to cycling, and the sample decomposed at 700 °C. In the image of the decomposed sample, some porous areas (circled) were detected which might be due to the decomposition of LiOH at high temperatures. Although the EDS graphs of this porous region did not show any impurity and were found to be composed of Cl (LiCl) and O (LiOH). Some contaminations (white spot circled in micrograph) were detected in the sample with zero thermal cycles as shown in Figure 9a. These contaminated elements possibly came from the refractory of the furnace during sample preparation while melting the two salts for homogeneity. Other than the small white spot, the EDS results of both of these samples were the same in which Cl represented LiCl and O represented LiOH and no other element was detected which proved that binary salt was in agreement with XRD results and chemically stable at high temperature.

### 3.6. X-ray Photoelectron Spectroscopy (XPS) Characterization

**Figure 10:**
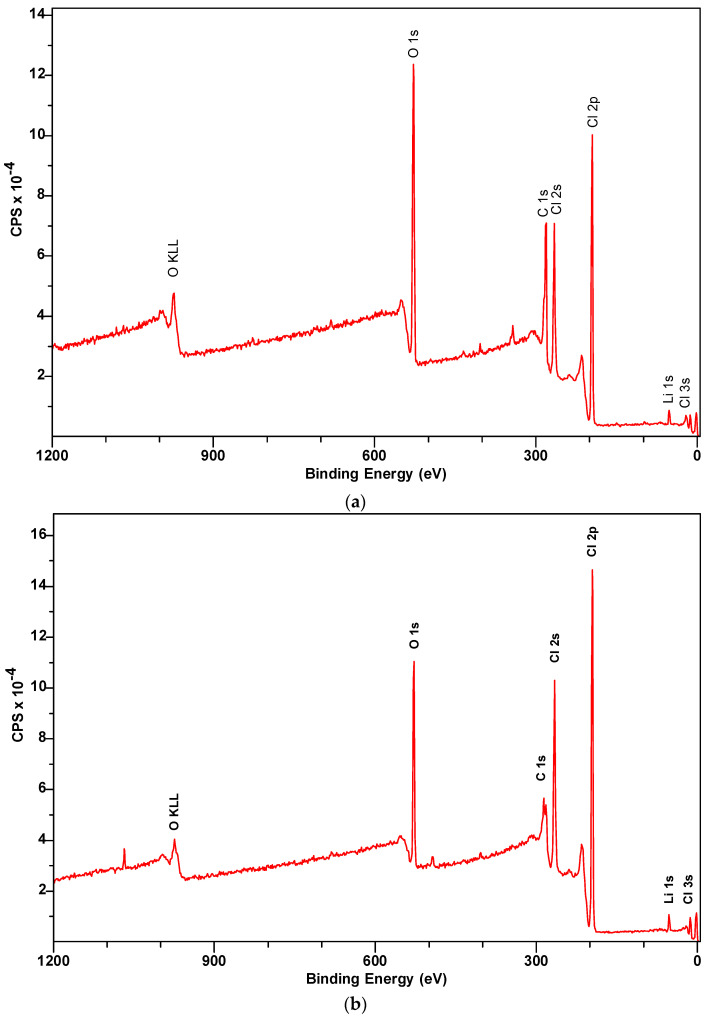
(**a**) XPS spectra of 32 mol% LiCl-68 mol% LiOH with zero thermal cycle. (**b**) XPS spectra of 32 mol% LiCl-68 mol% LiOH decomposed at 700 °C.

Similar to the microscopy experiments, to verify the chemical stability of the binary salt, 32 mol% LiCl-68 mol% LiOH with no cycling and decomposed at 700 °C was examined by XPS and displayed in Figure 10. Binary salt with no cycling and decomposed at 700 °C had no detectable impurity as shown in Figure 10a,b. In both the samples, O KLL, O 1s, C 1s, Cl 2s, Cl 2p, Cl 3s, and Li 1s were detected which were the desired elements for our sample except C 1s which has possibly been detected by the carbon tape. The binding energies and the atomic percentages of each element in the two samples are listed in Table 5. According to the NIST database, the binding energy of Li 1s is reported as 56 eV which indicated LiCl. Moreover, LiCl is shown for Cl 2p in the range of 193–199 eV binding energy and LiOH is reported at 530 eV for O 1s. After comparing the spectra of Li 1s, Cl 2p, and O 1s with the NIST database, Li 1s indicated LiCl, Cl 2p indicated LiCl, and O 1s indicated LiOH. Moreover, it can be seen that the peak intensity of the O 1s spectrum is larger in the sample with no heating and cooling cycle than the O 1s in the decomposed sample (compare Figure 10a,b). Also, the atomic percentage of O 1s is higher, which is 53.34% in the as-prepared sample than in the decomposed sample, which is 34.99%. This means that O 1s indicated the LiOH which decomposed at higher temperatures, and was later transformed into gas. A similar scenario is also observed for chlorine. While Cl 2p indicated LiCl, which showed a higher intensity and atomic percentage in the decomposed sample than in the sample with no heating/cooling cycle. This is due to the higher concentration of LiCl in the decomposed sample. Thus, it is clear from the above discussion that the binary salt is chemically stable up to 500 °C and is a potential candidate for thermal energy storage.

## 4. Conclusions

In this article, the thermophysical properties, thermal repeatability, and thermal decomposition behavior of the binary salt mixture with the appropriate compositions were investigated systematically. We considered 32 mol% LiCl-68 mol% a potential candidate because of its favorable thermal properties. The thermal/structural instability was verified using X-ray diffraction (XRD). The melting range determined for the chosen binary salt mixture was from 269 °C to 292 °C, and the heat of fusion was 379 kJ/kg, which are adequate values for thermal energy storage applications.

The binary salt mixture acquired good thermal repeatability when ran for 30 heating/cooling cycles with a negligible deviation in melting temperature. However, the heat of fusion values deviated slightly which might be due to the evolving gasses during heating, creeping, or the different impurities present in the sample. It also showed good thermal stability with a very minor weight loss of less than 1.5%, except during the first few cycles, indicating that the binary salt mixture is a potential candidate for energy storage.

The upper-temperature limit was recorded as 500 °C and from the XRD analysis, the observed minor peaks relating to lithium oxide in the sample decomposed at 700 °C, which could result from the decomposition of LiOH, as LiOH can be decomposed at a higher temperature. Other than that, there were negligible changes in the intensities of the peaks. SEM/EDS show good morphology and well-distributed elements, indicating good homogeneity of the binary salt mixture. The EDS of 32 mol% LiCl-68 mol% LiOH (as prepared and decomposed sample) showed good chemical stability without any detectable impurities present. However, there are some impurities detected that could result from the furnace during sample preparation. XPS spectra 32 mol% LiCl-68 mol% LiOH (as prepared and decomposed sample) showed the main peaks of elements Li 1s, Cl 2p, and O 1s, indicating LiCl and LiOH with no other impurities present, indicating excellent chemical stability. Thus, it is concluded that the chosen material is thermally stable and has the potential to be used for thermal energy storage.

## Figures and Tables

**Table 1:** Demand and consumption of lithium salts-Forecast 2011–2025 [21,23,24].

Year	Total Demand by Compound MT LCE	Total Consumption by Applications MT LCE
**2011**	140,056	129,282
**2015**	204,732	188,983
**2020**	327,743	302,532
**2025**	540,119	498,571

**Table 2:** List of binary salt mixtures investigated in the current study.

S. No	Samples
1	27 mol% LiCl-73 mol% LiOH
2	32 mol% LiCl-68 mol% LiOH
3	37 mol% LiCl-63 mol% LiOH
4	42 mol% LiCl-58 mol% LiOH

**Table 3:** Calibration of STA using gold and individual testing of lithium chloride.

Standard Material	Expected Melting Point °C	Measured Melting Point °C	Relative Error %	Expected Heat of Fusion J/g	Measured Heat of Fusion J/g	Relative Error %
**Gold (Au)**	1064.18	1061.61	0.2	64.6	67	3
**Lithium Chloride (LiCl)**	610	602	1	441	449	1

**Table 4:** Melting temperature and heat of fusion for four PCM samples.

S. No	Samples	Onset Temperature (Heating) °C	Onset Temperature (Cooling) °C	Heat of Fusion J/g	Heat of Solidification J/g
**1**	27 mol% LiCl-73 mol% LiOH	271	265	214	198
**2**	32 mol% LiCl-68 mol% LiOH	269	265	379	375
**3**	37 mol% LiCl-63 mol% LiOH	270	268	297	218
**4**	42 mol% LiCl-58 mol% LiOH	289	282	230	201

**Table 5:** Atomic percentages obtained by XPS of two samples (32 mol% LiCl-68 mol% LiOH with no cycle and decomposed at 700 °C).

Sample	Elements	Position eV	Atomic %
32 mol% LiCl-68 mol% LiOH (with zero cycles)	Li 1s	52	1.76
	Cl 2p	195	44.90
	O 1s	528	53.34
32 mol% LiCl-68 mol% LiOH (decomposed at 700 °C)	Li 1s	53	2.08
	Cl 2p	196	62.93
	O 1s	528	34.99

## Data Availability

Not applicable.
